# Decoding the Mitochondrial Genome of the Tiger Shrimp: Comparative Genomics and Phylogenetic Placement Within Caridean Shrimps

**DOI:** 10.3390/genes16040457

**Published:** 2025-04-16

**Authors:** Zhengfei Wang, Weijie Jiang, Jingxue Ye, Huiwen Wu, Yan Wang, Fei Xiong

**Affiliations:** 1 Jiangsu Key Laboratory for Bioresources of Saline Soils, Jiangsu Synthetic Innovation Center for Coastal Bio-Agriculture, Jiangsu Provincial Key Laboratory of Coastal Wetland Bioresources and Environmental Protection, School of Wetlands, Yancheng Teachers University, Yancheng 224001, China; 18252737019@163.com (W.J.); 18811930687@163.com (H.W.); 19732779680m@sina.cn (Y.W.); 2 College of Bioscience and Biotechnology, Yangzhou University, No. 48, Wenhui East Road, Hanjiang District, Yangzhou 225009, China; 3 School of Life Science, Northwest University, No. 229 Taibai North Road, Beilin District, Xi’an 710069, China; 15137695295@163.com

**Keywords:** mitochondrial genome, phylogenetic analysis, Decapoda, freshwater shrimp, Atyidae

## Abstract

Background/Objectives: Freshwater shrimps of the family Atyidae, particularly the hyperdiverse genus Caridina, are keystone decomposers in tropical aquatic ecosystems and valuable aquaculture resources. However, their evolutionary relationships remain unresolved due to conflicting morphological and molecular evidence. Here, we sequenced and characterized the complete mitochondrial genome of *Caridina mariae* (Tiger Shrimp), aiming to (1) elucidate its genomic architecture, and (2) reconstruct a robust phylogeny of Caridea using 155 decapod species to address long-standing taxonomic uncertainties. Methods: Muscle tissue from wild-caught *C. mariae* (voucher ID: KIZ-2023-001, Guangdong, China) was subjected to Illumina NovaSeq 6000 sequencing (150 bp paired-end). The mitogenome was assembled using MITObim v1.9, annotated via MITOS2, and validated by PCR. Phylogenetic analyses employed 13 protein-coding genes under Bayesian inference (MrBayes v3.2.7; 10^6^ generations, ESS > 200) and maximum likelihood (RAxML v8.2.12; 1000 bootstraps), with *Harpiosquilla harpax* as the outgroup. The best-fit substitution model (MtZoa + F + I + G4) was selected via jModelTest v2.1.10. Results: The 15,581 bp circular mitogenome encodes 37 genes (13 PCGs, 22 tRNAs, and 2 rRNAs) and an A + T-rich control region (86.7%). Notably, trnS1 lacks the dihydrouracil arm—a rare structural deviation in Decapoda. The 13 PCGs exhibit moderate nucleotide skew (AT = 0.030; GC = −0.214), while nad5, nad4, and nad6 show significant GC-skew. Phylogenomic analyses strongly support (PP = 1.0; BS = 95) a novel sister-group relationship between *Halocaridinidae* and *Typhlatyinae*, contradicting prior morphology-based classifications. The monophyly of *Penaeoidea*, *Astacidea*, and *Caridea* was confirmed, but Eryonoidea and Crangonoidea formed an unexpected clade. Conclusions: This study provides the first mitogenomic framework for *C. mariae*, revealing both conserved features (e.g., PCG content) and lineage-specific innovations (e.g., tRNA truncation). The resolved phylogeny challenges traditional Caridea classifications and highlights convergent adaptation in freshwater lineages. These findings offer molecular tools for the conservation prioritization of threatened *Caridina* species and underscore the utility of mitogenomics in decapod systematics.

## 1. Introduction

Decapoda, a group of crustaceans, is the most plentiful and diverse in terms of both quantity and prevalence. Decapoda includes shrimps, crabs, and hermit crabs [1,2]. Shrimps are widely distributed throughout the world, notably in the shallower regions of oceans. Due to their remarkable values both in commerce and ecology, shrimps have been extensively studied in the field of molecular biology [3].

In the Decapoda, Atyidae belongs to the Pleocyemata sub-order, and is present in both tropical and temperate habitats [4]. Atyidae encompasses approximately 400 species and forty genera [5]. The distinctive feature of the family Atyidae is the presence of bristled pincers that resemble hair and a diminutive body size. Notably, recent research has indicated that the taxonomic classification of Atyidae has been influenced by crustal movement [6]. The ancient Gangwa continental rift, in conjunction with the eventual closing off of the Tethyan Sea, is generally believed to be the primary factor contributing to the further divergence of this taxonomic group [7]. For the sake of performing a thorough study of molecular systematics, it is important to acquire the most comprehensive mitochondrial genome data of the Atyidae.

With more than 300 species, *Caridina* is distinguished for its rich diversity, predominantly in the Atyidae family within the Indo-West Pacific region [8,9,10]. Over one hundred distinct species of *Caridina* are widely distributed in China, such as Hunan, Yunnan, and Guangdong [11,12,13]. Among *Caridina*, *C. mariae* is special for its transparent body, yellow antennas, and uropodium. Additionally, the skin on the tail of *C. mariae* is similar to that of a tiger, so it is commonly known as “Tiger Shrimp”. *C. mariae* demonstrates a pronounced proclivity for exceptional water quality and a preference for thriving in acidic soft water [14,15,16,17,18]. Thus, *C. mariae* is frequently seen in tiny mountain streams. *C. mariae* also leans toward habitats abundant in aquatic vegetation or rocky terrains and prospers within a temperature range from 8 to 30 °C. Moreover, *C. mariae* underwent speciation, separating from *C. cantonensis* and becoming a separate species in 2014. *C. mariae* often forms hybrids with *C huananensis*, *C*. sp. “*Chinese Zebra*”, and *C*. cf. *serrata*. Furthermore, in certain geographical distributions, it is discovered in a symbiotic relationship with *C. conghuensis*. It is worth noting that *C. mariae* lived with *C. cantonensis* in the creek where it was originally been depicted [19,20,21]. The Atyidae family plays an important ecological role in the decomposition of sediments, the processing of particulate organic matter, and the formation of a benthic community [6]. These ecological and morphological features contrast with the unresolved molecular phylogeny of *Caridina*, necessitating complete mitochondrial genome data. Therefore, it is imminent to sequence more species for deep research.

Mitochondria, as semi-autonomous organelles [22], possess their separate genome and exhibit a marked level of conservatism in several aspects like size, composition, and structure [23]. Compared to other genes, the mitochondrial genes in metazoans have a higher degree of conservation [24]. Since mitochondrial genes diverge from nuclear genes in that they have more copies per cell, a pattern of maternal inheritance, and a higher rate of mutation than nuclear genes, these genes have gained popularity as a preferred focus in research on the evolutionary history and geographic distribution of multicellular organisms [25].

The purpose of this study was to sequence and characterize the mitochondrial genome of *C. mariae*. In particular, we investigated the genetic composition of mitochondria, nucleotide usage in *C. mariae*, codon composition in protein-coding genes, and a detailed analysis of tRNA secondary structures. Ultimately, the phylogenetic positions of *C. mariae* were established using nucleotide genes and protein-coding sequences for 155 species.

## 2. Materials and Methods

### 2.1. Sampling, DNA Extraction, and Sequencing

The samples were obtained from the aquatic market in Guangzhou (Guangdong province, China). The strategy adopted for molecular species identification involved amplifying the conserved *Cox1* and *16S rRNA* and comparing the obtained sequences with those annotated by Genbank. Sequence homology was verified using BLAST (https://blast.ncbi.nlm.nih.gov/, accessed on 9 October 2023) against the GenBank nucleotide database. To achieve complete lysis, the samples were subjected to a temperature of 55 °C for five hours. After this, the whole DNA samples were acquired from the muscle tissue with the Aidlab genomic DNA extraction kit (Aidlab Biotech, Beijing, China). Following the manufacturer’s suggestion, genomic DNA was eluted using a volume of fifty milliliters of double-distilled water (ddH_2_O). The quality of the extracted DNA samples was assessed using electrophoresis, and then, the DNA samples were stored at −20 °C until PCR amplification. The mitogenomes of *C. mariae* were sequenced by next-generation sequencing (Illumina HiSeq 4000; Shanghai Origingene Bio–pharm Technology Co., Ltd., Shanghai, China) [26,27,28,29,30,31].

### 2.2. Sequence Analysis and Gene Annotation

After being assembled, revised, and annotated, the sequences were uploaded to GenBank with the accession number PQ359442. To search for genomic sequences related to mitosis, it is recommended to use the BLAST tool available on the NCBI website. The thirteen PCGs (protein-coding genes) were initially identified using the “ORF (Open Reading Frame) Finder” tool provided by NCBI and the MITOS website. tRNA secondary structures were predicted using the MITOS web server (http://mitos2.bioinf.uni-leipzig.de/index.py, accessed on 10 October 2023) with default parameters. This was conducted to determine the specific mitochondrial genetic code of invertebrates. The determination of potential stem-loop secondary structures in these tRNA gene sequences was performed using the MITOS online tool [32]. The software MEGA 11.0 was utilized for analyzing the nucleotide composition and computing the relative synonymous codon usage (RSCU). RSCU (relative synonymous codon usage) was determined by the use of PCGs, and any absent codons were excluded from this procedure. The component skew analysis was conducted using the following equations: AT-skew (Adenine-Thymine skew) = (A − T)/(A + T) and GC-skew (Guanine-Cytosine skew) = (G − C)/(G + C), with both formulations being integral to the procedure. The web-based program, Organellar Genome DRAW, was employed to accurately map the mitochondrial genome by presenting genetic information. The circular mitogenome map was generated using OGDRAW (https://chlorobox.mpimp-golm.mpg.de/OGDraw.html, accessed on 11 October 2023) with manual curation.

### 2.3. Phylogenetic Analysis

The taxonomic placement of *C. mariae* within the Decapoda was assessed by utilizing phylogenetic tree reconstruction as the basis for the research. A phylogenetic reconstruction was conducted utilizing the data from 13 PCGs of all the shrimp species available in GenBank. Additionally, the mitogenomes of 153 species from nine distinct superfamilies were included, in addition to the recently sequenced mitogenome of *Candida mariae*. The research encompassed the superfamilies *Sergestoidea*, *Penaeoidea*, *Eryonoidea*, Crangonoidea, *Astacidea*, *Palinuroidea*, *Sergestoidea*, *Stenopodidea*, and *Caridea*, with the addition of *H. harpax* (AY699271.1) as the outgroup. A comparison was conducted between the nucleotide sequences of each gene and the resulting amino acid sequences using MUSCLE 3.8 in MEGA 11.0. Following the alignment of 13 PCGs from 153 mitochondrial genomes using the default settings, the amino acid sequences were concatenated for further analysis. The sequences were arranged in a cascade to aid in phylogenetic analysis. MrBayes v3.2.6 and RaxML were employed to calculate Bayesian inference (BI) and maximum likelihood (ML), respectively. The optimal model for assessing amino acid similarities was identified using jModeltest. The best-fit substitution model (MtZoa + F + I + G4) was selected based on Akaike Information Criterion (AIC) in jModeltest v2.1.10 [33]. During two simultaneous runs, a BI analysis was conducted on the matrix for a total of ten quadrillion generations. Conducting two experiments simultaneously allowed for the interchange of Markov chain Monte Carlo (MCMC) chains, with a combined total of four chains (comprising three hot chains and one cold chain), each lasting 1000 generations. The tests were conducted to promote the exchange. To assess the convergence of collected parameters and potential autocorrelation, we relied on the software Tracer 1.6 (http://tree.bio.ed.ac.uk/software/tracer/, accessed on 12 October 2023), and all the metrics had an effective sample size (ESS) exceeding 200. Chain convergence was further validated using Tracer v1.6 (http://beast.community/tracer, accessed on 12 October 2023) with ESS thresholds > 200 for all the parameters. In addition, we analyzed the average standard deviation of the split frequency between the two runs and confirmed it was below 0.01. After removing the initial twenty-five percent of trees, known as the “burn-in” stage, Bayesian posterior probabilities were estimated based on the fifty percent majority-rule consensus of the post-burn-in trees that were sampled before reaching a stable state. The resulting phylogenetic trees obtained were visualized using FigTree version 1.4.2.

## 3. Result

### 3.1. Genome Organization and Base Composition

**Figure 1:**
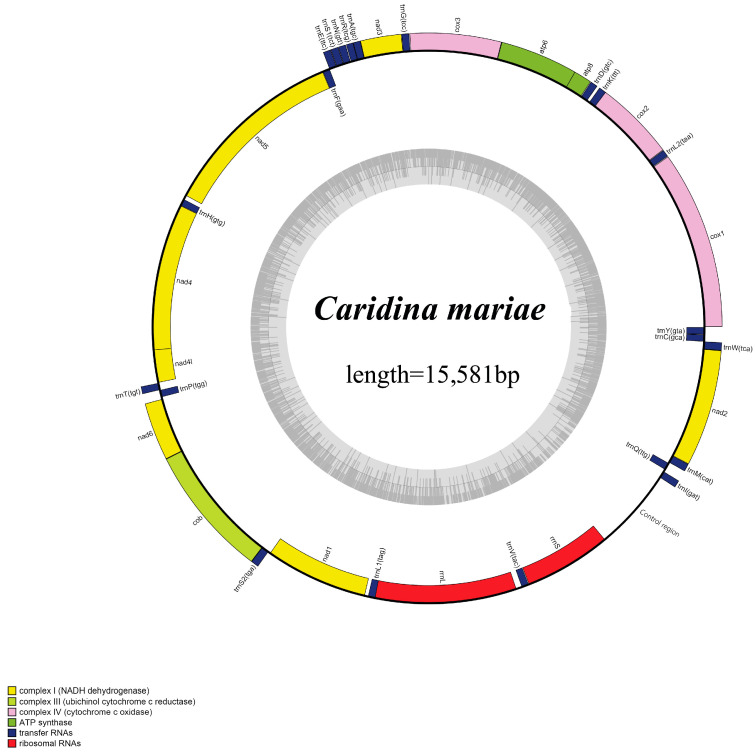
Circular mitogenic chart from *C. mariae*. The coding of proteins, ribosomal genes, and tRNAs are represented using standard abbreviations.

The total length of the mitochondrial genome in *C. mariae* was 15,581 bp (Figure 1), falling between 15,550 bp (*C. gracilipes*) and 16,430 bp (*Typhlatya galapagensis*) in the Atyidae family. There were a total of 38 genes with 14 genes positioned on the negative strand, while a larger proportion of 24 genes were situated on the positive strand. These genes consisted of 13 PCGs, 22 tRNAs, 2 rRNAs, and 1 control region (CR) (Table 1). The genetic configuration of this species was in harmony with the standard genetic makeup of a normal metazoan.

The total mitochondrial genome of *C. mariae* exhibited a notably high A + T content of 68.9%, as shown in Table 2 through the base composition analysis. In Decapoda, the A + T content varied from 58.8% to 77.4%, while in *Caridea*, it ranged from 58.8% to 70.2% in PCGs. The A + T content was 67.1% in tRNAs, 68.1% in rRNA, and 86.7% in CR. A positive AT bias of 0.030 and a negative GC bias of −0.214 were observed in the entire sequence. The 13 PCGs exhibited a small overall GC-skew of 0.007. However, *nad5*, *nad4*, *nad4L*, and *nad6* manifested a noticeable bias towards GC-skew, as depicted in Table 2.

The base composition showed that the content of A + T was high (68.9%) in the complete mitogenome of *C. mariae* (Table 2), and ranged from 58.8 to 77.4% in Decapoda and 58.8–70.2% in *Caridea*, PCGs (67.1%), tRNAs (68.1%), rRNA (72.9%), and CR (86.7%) [34].

### 3.2. Protein-Coding Genes and Non-Coding Regions

The cumulative length of 13 PCGs was 11,128 bp, representing 71.4% of the whole mitochondrial genome. The sizes of 13 PCGs varied from 159 bp for *atp8* to 1692 bp for *nad5*, as outlined in Table 1 and Table 2. In Table 1, four PCGs were encoded on the heavy chain, whereas the remaining PCGs (*cox1*, *cox2*, *cox3*, *atp8*, *atp6*, *nad2*, *nad3*, *nad6*, and *cob*) were encoded on the light chain. The start codon ATG was used by six genes (*atp6*, *cob*, *cox2*, *cox3*, *nad4*, and *nad4l*). The gene *cox1* utilized the start codon CAA. Genes like *atp8*, *nad3*, *nad2*, and *nad5* consistently began with the start codon ATT, while *nad6* and *nad1* showed sporadic use of the codon ATA. Out of the ten PCGs, it was discovered that they concluded with the common stop codon TAA, except for the *cob* and *nad1* genes, which were noted to have the stop codon TAG as their termination codon.

**Figure 2:**
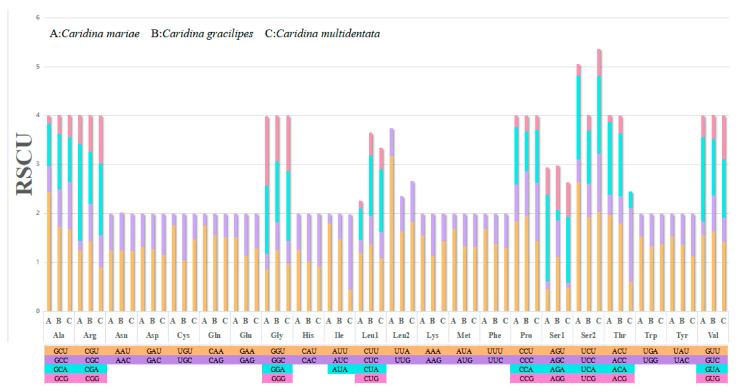
Codon usage of three rice shrimp species (A: *C. mariae*, B: *C. gracilipes*, and C: *C. multidentata*).

Within the mitochondrial genome of *C. mariae*, the codon number and the proportional usage of synonymous codons were documented (Table 3). Codon use patterns for 13 PCGs are illustrated in Figure 2. A study was implemented to determine the relative synonymous codon usage (RSCU) and abundance of the crypto family PCGs in *C. mariae* and two additional species of *Caridina*. Five RSCUs were utilized by *C. mariae* the most frequently: *Ser2*, *Thr*, *Arg*, *Pro*, and *Val*. The frequency of utilization was essentially the same across all three species of *Caridina*, except *Leu*, *Ser2*, and *Thr* which were not used. From what can be observed, the third codon bit favors the A/T combination.

Similarly to other invertebrate mitochondrial DNA, there were instances where the genes overlapped and contained non-coding nucleotides. In total, 16 intergenic spacers, ranging in size from 1 to 50 bp, were identified. Measuring 50 bp, the largest intergenic spacer was located between the *rrnL* and *trnV* genes. Twelve genes shared a common region of 1–40 bp. The greatest 40 bp region was pinpointed as being positioned between *trnL1* and *rrnL*, as depicted in Table 1. Significantly, the *trnH* sequence of *C. mariae* was completely included within the *nad4* region. (Table 1). While total overlap was observed in certain viruses [35], it remained uncommon in mammals. The biggest non-coding area of *C. mariae* was determined to be the putative regulatory region.

### 3.3. Transfer and Ribosomal RNA Genes

**Figure 3:**
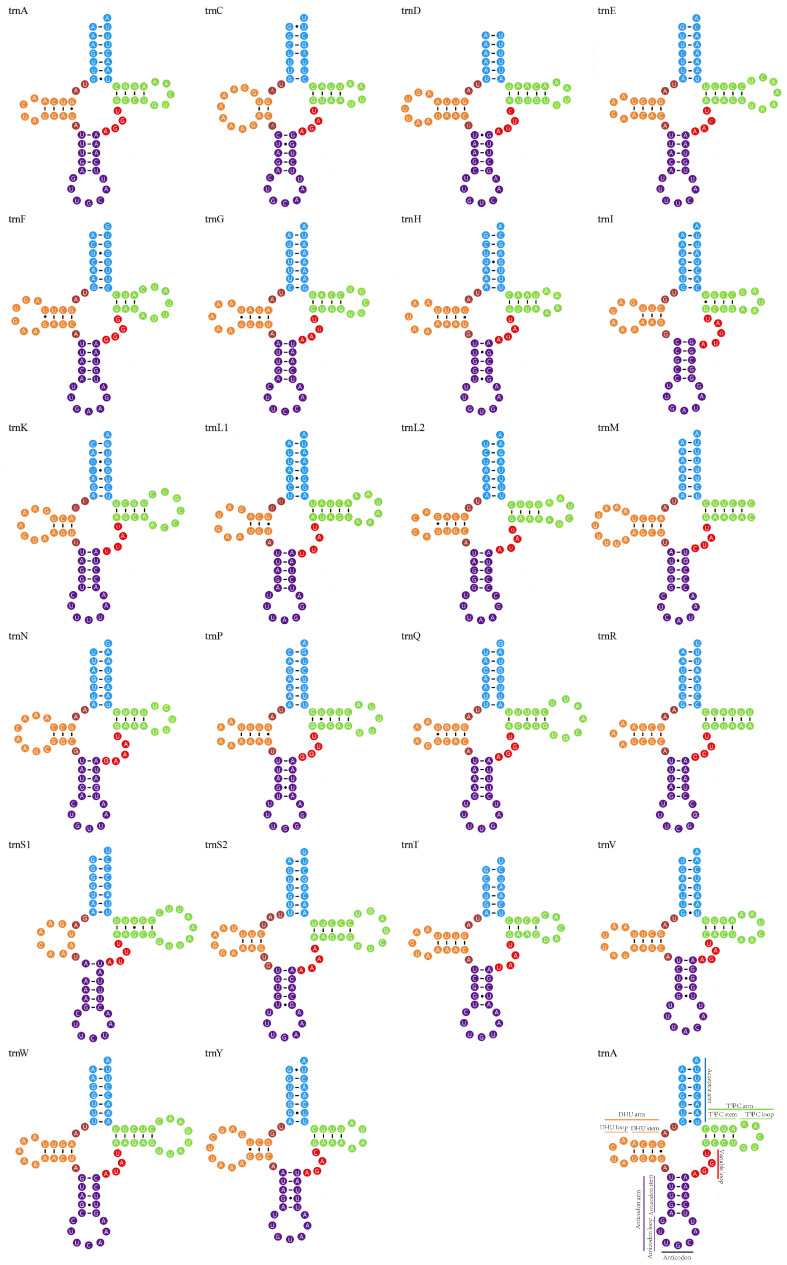
Putative secondary structures of *C. mariae* mitochondrial genomes. The tRNAs are labeled with corresponding amino acid abbreviations.

The mitochondrial genome of *C. mariae* encoded 22 tRNA genes, each of which was predicted to fold into the secondary structure of the leaves of cloverleaf. The tRNAs varied in size from 62 bp (*trnR*) to 70 bp (*trnW* and *trnS2*) (Table 1). This could be attributed to evolutionary rate variation. Additionally, it was worth mentioning that tRNAs contain a multitude of basic mismatches, including 31 GU mismatches, 1 CC mismatch, 2 UU mismatches, 2 CA mismatches, and 3 UC mismatches (Figure 3).

The cumulative length of the 22 tRNA genes in the mitochondrial genome of *C. mariae* was 1455 bp. The combined A + T content of the tRNA gene was 68.1%, which closely resembled that of the other *Caridina* species (Table 2). The mitochondrial RNA (mtRNA) exhibited an AT-skew of 0.015 and a stronger GC-skew of 0.125. There were a total of 8 tRNA genes (*trnF*, *trnH*, *trnP*, *trnL1*, *trnV*, *trnQ*, *trnC*, and *trnY*) identified on the heavy strand, and 14 tRNA genes (*trnL2*, *trnK*, *trnD*, *trnG*, *trnA*, *trnR*, *trnN*, *trnS1*, *trnE*, *trnT*, *trnS2*, *trnI*, *trnM*, and *trnW*) identified on the light strand. The *12S rRNA* gene was positioned between *trnL1* and *trnV*, while the *16S rRNA* gene was positioned between *trnV* and the presumed regulatory region. The β chains encoded both rRNA genes. In line with the mitochondrial genetic makeup observed in various other shrimp varieties, the mitochondrial genetic composition of *C. mariae* included the *16S rRNA* and *12S rRNA* genes with lengths measuring 1326 bp and 797 bp, correspondingly. The combined A + T content of the two rRNA genes was 72.9%. Moreover, the A + T skew value was negative (−0.070, Table 2).

### 3.4. Phylogenetic Relationship

**Figure 4:**
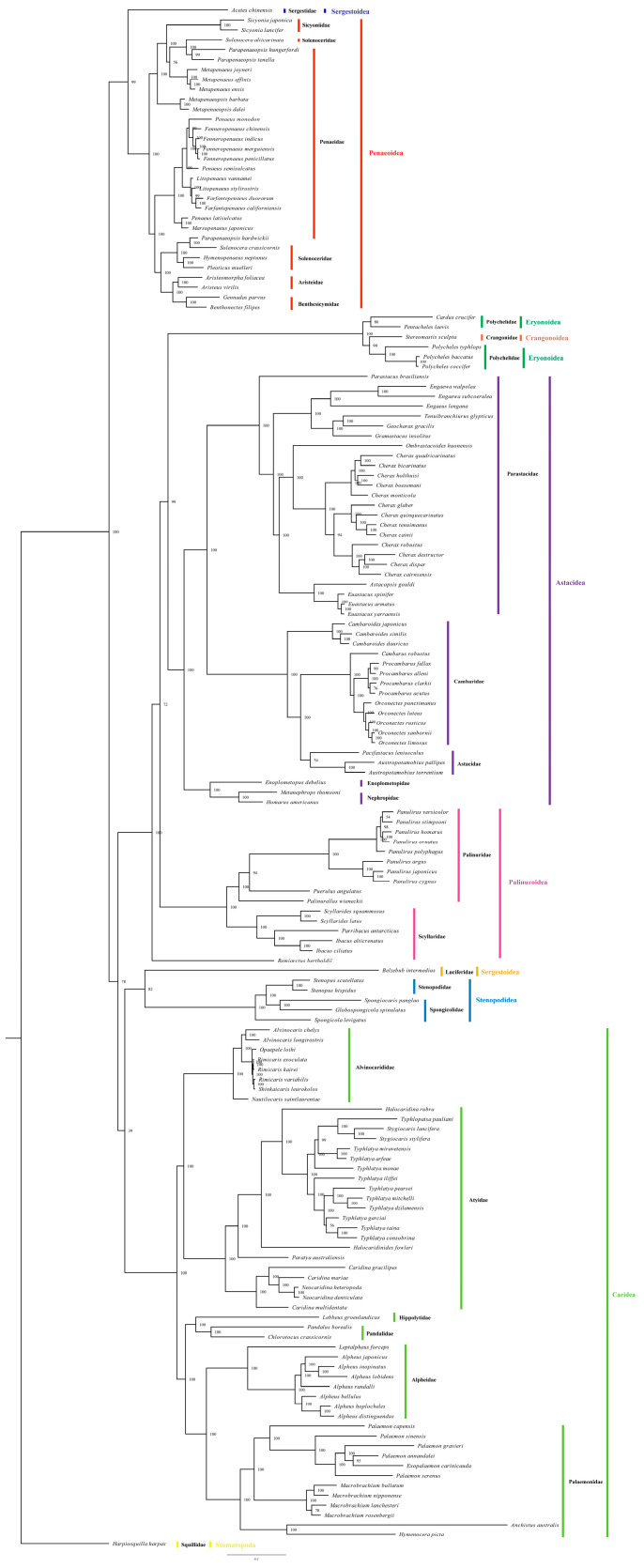
Phylogeny of *C. mariae* based on amino acid sequences. The phylogenetic tree was inferred from the amino acid sequences of 13 mitogenome PCGs using BI methods. Numbers on branches indicate posterior probability (BI).

With *H. harpax* as the outgroup species (Figure 4), this study utilized the BI approach and ML method to analyze a total of 155 species. The species of *Dendrobranchiata* and *Pleocyemata* have been chosen. The study effectively showcases the BI tree graph by relying heavily on the supporting values provided. The taxonomic classification was as follows: (*Sergestoidea* + *Penaeoidea*) + ((*Eryonoidea* + *Crangonoidea*) *Astacidea* + *Palinuroidea*) + (*Sergestoidea* + *Stenopodidea*) *Caridea*. *Penaeoidea*, *Astacidea*, *Stenopodidea*, and *Caridea* exhibited clear monophyletic grouping, but *Eryonoidea* and *Crangonoidea* had a syngeneic association. The phylogenetic relationship of *Caridea* was primarily categorized into two main groups: (*Alvinocarididae* + *Atyidae*) and (*Hippolytidae* + *Pandalidae* + (*Alpheidae* + *Palaemonidae*)). Within the *Atyinae* subfamily, there was a larger branch that included *Caridina* and *Neocaridina*, both of which were freshwater shrimp species. Other subfamilies such as *Typhlatyinae* (*Stygiocaris*, *Typhlatya*, and *Typhlopatsa*), *Caridellinae* (*Halocaridina rubra*), and *Paratyinae* (*Paratyaaustraliensis*) were grouped as sister groups. The results were consistent with previous studies [20]. What set this study apart was the identification of a close relationship between *Halocaridinides* and *Typhlatyinae*, a departure from earlier studies [36]. The *Atyinae* species likely originated during the early Permian to Jurassic period, with the subsequent evolution leading to the development of their modern characteristics, as evidenced by the analysis of their mitochondrial genome structure and phylogenetics. Additionally, exploring mitogenome phylogenetics in the *Palaemon* genus of Crustacean shed light on the hidden variation within the species *Palaemon elegans*.

## 4. Discussion

The length of the mitochondrial genome in *C. mariae* was measured to be 15,581 bp. The mitogenome of *C. mariae* exhibits high conservation and shares close similarities with congeneric species and their common ancestor [11,12,13]. A systematic investigation revealed that *Caridea* mostly occurs in the evolutionary linkage of (*Alvinocarididae* + *Atyidae*) + ((*Hippolytidae* + *Pandalidae*) + (*Alpheidae* + *Palaemonidae*).

The mitochondrial genome of *C. mariae* exhibited the characteristic *Atyidae* architecture, consisting of 13 protein-coding genes (PCGs), 22 tRNAs, and 2 rRNAs flanked by a control region (CR), with 12 overlapping regions and 16 non-coding spacers identified in its gene arrangement—features that reflect the substantial genomic changes accumulated since this lineage diverged from its Permian–Jurassic progenitor, ultimately leading to the distinct characteristics seen in modern *Atyinae* species. Recent advances in livestock genomics reveal that long-term adaptation to human-driven selection pressures parallels key evolutionary patterns observed in wild decapods—both systems show how core genetic stability (e.g., conserved PCGs) can coexist with structural innovation (e.g., tRNA rearrangements), suggesting universal principles of genomic resilience under environmental challenges [37]. Gene overlap occurred in 12 locations, with 16 areas identified between the genes. *Ser2*, *Thr*, *Arg*, *Pro*, and *Val* stood out as the top relative synonymous codon usage (RSCU) values for tiger shrimp. Among these, the A/T nucleotide pairing was highlighted as the preferred choice for the third codon position. Similarly to the patterns in domestic livestock mtDNA studies—where conserved gene content coexists with lineage-specific structural adaptations—our findings in *Caridina* freshwater shrimps demonstrate how mitochondrial architecture can simultaneously preserve core functions while evolving specialized features in response to ecological pressures, suggesting deep evolutionary parallels between vertebrate and invertebrate mitochondrial evolution under selection [38].

The Atyinae species probably originated from a common ancestor from the early Permian to Jurassic era. The phylogenetic analysis of mitogenome in the *Palaemon* provides insights into the hidden variation within the species *P. elegans*.

Freshwater shrimps play a crucial role in tropical and subtropical freshwater ecosystems, where they are either taken from the wild or cultivated for food [39]. As ecosystem engineers, they significantly influence sediment bioturbation and organic matter decomposition rates, maintaining water quality in their habitats. Among the 38 documented *Caridea* families, merely 7 contain true freshwater-adapted species, highlighting the evolutionary rarity of this ecological transition [40]. *Caridina* species belong to freshwater shrimps, which have considerable economic benefits and attractive quality. However, climate change-induced habitat fragmentation and invasive species competition now threaten over 30% of the endemic *Caridina* populations across Southeast Asia. The categorization position of freshwater shrimps is advantageous for the development of the aquaculture industry. This study employed a wide range of taxa samples for phylogenetic analysis, which provided significant information for the evolutionary analysis of *Caridina*.

## Figures and Tables

**Table 1:** Mitochondrial genomes of the *C. mariae*. Arrangement and notation.

Gene	Direction	Location	Size (bp)	Anticodon	Start Codon	Stop Codon	Intergenic Nucleotides
cox1	+	1–1533	1533		CAA	TAA	2
trnL2	+	1536–1599	64	TAA			1
cox2	+	1601–2308	708		ATG	TAA	−20
trnK	+	2289–2355	67	TTT			22
trnD	+	2378–2442	65	GTC			2
atp8	+	2445–2603	159		ATT	TAA	−7
atp6	+	2597–3271	675		ATG	TAA	−1
cox3	+	3271–4056	786		ATG	TAA	3
trnG	+	4060–4124	65	TCC			0
nad3	+	4125–4478	354		ATT	TAA	−2
trnA	+	4477–4540	64	TGC			−1
trnR	+	4540–4601	62	TCG			3
trnN	+	4605–4671	67	GTT			0
trnS1	+	4672–4738	67	TCT			0
trnE	+	4739–4806	68	TTC			−2
trnF	−	4805–4870	66	GAA			0
nad5	−	4871–6562	1692		ATT	TAA	36
trnH	−	6599–6662	64	GTG			0
nad4	−	6663–8001	1339		ATG	T(AA)	−7
nad4L	−	7995–8294	300		ATG	TAA	2
trnT	+	8297–8361	65	TGT			0
trnP	−	8362–8427	66	TGG			17
nad6	+	8445–8945	501		ATA	TAA	−1
cob	+	8945–10,081	1137		ATG	TAG	−2
trnS2	+	10,080–10,149	70	TGA			18
nad1	−	10,168–11,106	939		ATA	TAG	30
trnL1	−	11,137–11,203	67	TAG			−40
rrnL	−	11,164–12,489	1326				50
trnV	−	12,540–12,606	67	TAC			2
rrnS	−	12,609–13,405	797				0
CR		13,406–14,151	746				0
trnI	+	14,152–14,216	65	GAT			19
trnQ	−	14,236–14,303	68	TTG			6
trnM	+	14,310–14,377	68	CAT			0
nad2	+	14,378–15,382	1005		ATT	TAA	−2
trnW	+	15,381–15,450	70	TCA			−1
trnC	−	15,450–15,513	64	GCA			0
trnY	−	15,514–15,579	66	GTA			2

**Table 2:** Composition of nucleotides and asymmetry of the mitochondrial genome of *C. mariae*.

*C. mariae*	Size (bp)	T(U) (%)	C (%)	A (%)	G (%)	A + T (%)	AT-Skew	GC-Skew
Mitogenome	15,581	33.4	18.9	35.5	12.2	68.9	0.030	−0.214
PCGs	11,128	39.6	16.3	27.4	16.6	67.1	−0.182	0.007
cox1	1533	35.5	18.3	28.0	18.2	63.5	−0.118	−0.004
cox2	708	33.1	20.1	32.8	14.1	65.8	−0.004	−0.174
atp8	159	39.6	20.1	33.3	6.9	73.0	−0.086	−0.488
atp6	675	38.1	18.5	28.9	14.5	67.0	−0.137	−0.121
cox3	786	35.9	19.2	29.1	15.8	65.0	−0.104	−0.098
cob	354	40.1	20.1	28.8	11.0	68.9	−0.164	−0.291
nad5	1692	41.5	11.6	26.1	20.7	67.7	−0.228	0.283
nad4	1339	43.6	11.0	24.4	21.0	68.0	−0.282	0.313
nad4l	300	44.3	8.7	26.3	20.7	70.7	−0.255	0.409
nad3	501	40.7	21.6	29.1	8.6	69.9	−0.166	−0.430
nad1	1137	38.5	19.3	26.9	15.3	65.4	−0.177	−0.115
nad6	939	44.9	11.2	25.1	18.7	70.1	−0.283	0.253
nad2	1005	40.4	21.4	27.7	10.5	68.1	−0.187	−0.340
tRNAs	1455	33.5	14.0	34.6	17.9	68.1	0.015	0.125
rRNAs	2123	39.0	9.2	33.9	17.9	72.9	−0.070	0.323
Control region	746	39.7	7.0	47.1	6.3	86.7	0.085	−0.051

**Table 3:** Number of codons and relative use of synonymous codons in the mitochondrial genomes of *C. mariae*.

Codon	Count	RSCU	Codon	Count	RSCU	Codon	Count	RSCU	Codon	Count	RSCU
UUU(F)	258	1.69	UCU(S)	115	2.63	UAU(Y)	104	1.53	UGU(C)	37	1.76
UUC(F)	47	0.31	UCC(S)	21	0.48	UAC(Y)	32	0.47	UGC(C)	5	0.24
UUA(L)	305	3.17	UCA(S)	75	1.71	UAA(*)	10	1.67	UGA(W)	75	1.53
UUG(L)	55	0.57	UCG(S)	10	0.23	UAG(*)	2	0.33	UGG(W)	23	0.47
CUU(L)	115	1.19	CCU(P)	70	1.84	CAU(H)	51	1.26	CGU(R)	19	1.25
CUC(L)	26	0.27	CCC(P)	29	0.76	CAC(H)	30	0.74	CGC(R)	3	0.2
CUA(L)	62	0.64	CCA(P)	44	1.16	CAA(Q)	71	1.75	CGA(R)	30	1.97
CUG(L)	15	0.16	CCG(P)	9	0.24	CAG(Q)	10	0.25	CGG(R)	9	0.59
AUU(I)	271	1.8	ACU(T)	102	1.97	AAU(N)	76	1.25	AGU(S)	20	0.46
AUC(I)	30	0.2	ACC(T)	21	0.41	AAC(N)	46	0.75	AGC(S)	7	0.16
AUA(M)	169	1.69	ACA(T)	77	1.49	AAA(K)	59	1.55	AGA(S)	77	1.76
AUG(M)	31	0.31	ACG(T)	7	0.14	AAG(K)	17	0.45	AGG(S)	25	0.57
GUU(V)	100	1.56	GCU(A)	152	2.44	GAU(D)	53	1.31	GGU(G)	54	0.86
GUC(V)	18	0.28	GCC(A)	33	0.53	GAC(D)	28	0.69	GGC(G)	20	0.32
GUA(V)	110	1.72	GCA(A)	54	0.87	GAA(E)	54	1.52	GGA(G)	87	1.39
GUG(V)	28	0.44	GCG(A)	10	0.16	GAG(E)	17	0.48	GGG(G)	89	1.42

## Data Availability

Mitochondrial genomes sequenced and de novo assembled in the current study were submitted to the NCBI GenBank database under the accession number PQ359442.
